# Automatic pulmonary fissure detection and lobe segmentation in CT chest images

**DOI:** 10.1186/1475-925X-13-59

**Published:** 2014-05-07

**Authors:** Shouliang Qi, Han J W van Triest, Yong Yue, Mingjie Xu, Yan Kang

**Affiliations:** 1 Sino-Dutch Biomedical and Information Engineering School, Northeastern University, Shenyang, China; 2 Key Laboratory of Medical Imaging Computing (Ministry of Education), Northeastern University, Shenyang, China; 3 Department of Radiology, Shengjing Hospital of China Medical University, Shenyang, China

**Keywords:** Lung, Pulmonary fissure, Lobe, CT, Segmentation, Computed-aided diagnosis

## Abstract

**Background:**

Multi-detector Computed Tomography has become an invaluable tool for the diagnosis of chronic respiratory diseases. Based on CT images, the automatic algorithm to detect the fissures and divide the lung into five lobes will help regionally quantify, amongst others, the lung density, texture, airway and, blood vessel structures, ventilation and perfusion.

**Methods:**

Sagittal adaptive fissure scanning based on the sparseness of the vessels and bronchi is employed to localize the potential fissure region. Following a Hessian matrix based line enhancement filter in the coronal slice, the shortest path is determined by means of Uniform Cost Search. Implicit surface fitting based on Radial Basis Functions is used to extract the fissure surface for lobe segmentation. By three implicit fissure surface functions, the lung is divided into five lobes. The proposed algorithm is tested by 14 datasets. The accuracy is evaluated by the mean (±S.D.), root mean square, and the maximum of the shortest Euclidian distance from the manually-defined fissure surface to that extracted by the algorithm.

**Results:**

Averaged over all datasets, the mean (±S.D.), root mean square, and the maximum of the shortest Euclidian distance are 2.05 ± 1.80, 2.46 and 7.34 mm for the right oblique fissure. The measures are 2.77 ± 2.12, 3.13 and 7.75 mm for the right horizontal fissure, 2.31 ± 1.76, 3.25 and 6.83 mm for the left oblique fissure. The fissure detection works for the data with a small lung nodule nearby the fissure and a small lung subpleural nodule. The volume and emphysema index of each lobe can be calculated. The algorithm is very fast, e.g., to finish the fissure detection and fissure extension for the dataset with 320 slices only takes around 50 seconds.

**Conclusions:**

The sagittal adaptive fissure scanning can localize the potential fissure regions quickly. After the potential region is enhanced by a Hessian based line enhancement filter, Uniform Cost Search can extract the fissures successfully in 2D. Surface fitting is able to obtain three implicit surface functions for each dataset. The current algorithm shows good accuracy, robustness and speed, may help locate the lesions into each lobe and analyze them regionally.

## Introduction

Worldwide, chronic respiratory diseases, such as Chronic Obstructive Pulmonary Disease (COPD), are a major cause of premature deaths in adults [1]. COPD alone, accounts for 4 million deaths annually, and is the third leading cause of death in the United States [2]. The early and accurate identification of chronic respiratory diseases is essential for their prevention and control.

Multi-detector Computed Tomography (CT) has become an effective and invaluable tool for the diagnosis of chronic respiratory diseases. Using modern CTs, within one breath hold the lung can be imaged resulting in several hundreds of high-resolution and near-isotropic sections with thicknesses of approximate 0.5 mm [3]. Based on these images, advanced techniques of image processing can quantitatively assess the volumes of the lung [4], the characteristics of lung cancer [5], the structures of airway tree[6,7] and blood vessel [8], and the size of emphysema-like region [9], and help study human lungs from both structural and functional viewpoints [10].

With the arrival of more precise diagnosis and treatment, it is essential to segment the lung into its constituent regions, or lobes, which are separated by fissures. In non-pathological cases, pulmonary fissures are the double layers of infolded invaginations of visceral pleura, and exit between the different lobes. In the left lung, the oblique fissure separates the lower lobe from the upper lobe, whereas in the right lung, the oblique and horizontal fissures separate the lower lobe from the upper and middle lobes respectively. Once the lobe is extracted accurately, one can regionally characterize and quantify, amongst others, the lung density, texture, airway structure, blood vessel structure, ventilation and perfusion. For the diagnosis of pulmonary emphysema for example, the volume, emphysema volume (EV), emphysema index (EI) and mean density can be specified for each lobe, which facilitates preoperative planning and postoperative evaluation of lung-volume reduction surgery [11].

Segmentation of lung lobes from the chest CT images is a grand challenge for several reasons. First of all, the normal fissures are about 1–3 mm thick, and have a density near that of the soft tissue, which makes it hard to see the full stretch of the fissure. Secondly, the appearances of the fissures exhibit a large range of natural variations, and may be incomplete or even absent and distorted by various diseases. Furthermore, different CT protocols may lead to different appearances of the fissure [12]. For conventional CT, the fissures are visualized as lucent bands devoid of vascularity, whereas they appear as sharp lines for high-resolution CT [13].

The segmentation of the individual lung lobes is an extensively studied topic. Two classes of algorithms are available; the first group only uses the appearances of the fissures, while the other also utilizes further anatomical information from the lung, bronchus, and vessel structures. A brief summary of the model, feature and extraction methods in literature is presented in Table 1, and further details are given below.

**Table 1:** Selected algorithm and used models, features and extraction scheme

**Authors**	**Models**	**Features**	**Extraction scheme**
*Algorithms using fissure appearances*			
Pu et al. [14-16]	▪ The surface shaped structure	▪ Marching cubes algorithm, Laplacian smoothing and extended Gaussian image	▪ Implicit surface fitting using Radial Basis Functions (RBF)
Rikxoort et al. [17]	▪ Difference with the other texture	▪ Trained features	▪ Supervised filter and classier
Wei et al. [18]	▪ A curvilinear line in 2D slice	▪ Line structure	▪ Adaptive fissure sweeping and wavelet transform
Ross et al. [19,20]	▪ Ridge-like structure in 2D slice	▪ Ridgeness	▪ Thin plate splines and maximum a posteriori estimation
Wang et al. [21,22]	▪ Smooth high-intensity curve in 2D slice	▪ Intensity or ridgeness	▪ A curve growing algorithm modeled by Bayesian network
*Algorithms using lung, bronchus, and vessel information*			
Zhang et al. [23]	▪ Smooth surface	▪ Ridgeness image	▪ Fuzzy reasoning system
	▪ Ridge-like structure in 2D slice	▪ Anatomic pulmonary atlas	
Ukil et al. [24]	▪ Sparseness of the vessel	▪ Ridgeness	▪ 3D watershed transform
	▪ Match with bronchus tree structure		▪ Optimal surface
	▪ Ridge-like structure in 2D slice		
Rikxoort et al. [25-27]	▪ The lung borders	▪ Trained features for fissure	▪ Supervised filter
	▪ Airways and fissures		▪ Registration
Wei et al. [28]	▪ Different texture for fissure	▪ Texture analysis	▪ Dynamic programming
	▪ Large continuous fissure surface	▪ Projection	
Kuhnigk et al. [29], Lassen et al. [30]	▪ Sparseness of the vessel	▪ The original data removed blood vessel	▪ Cost image
	▪ High intensity	▪ The vasculature	▪ Multi-dimensional interactive watershed transform
	▪ Match with bronchus tree structure	▪ The bronchial tree	
	▪ Separation by surface-shaped fissure	▪ Pulmonary fissures	
Appia et al. [31]	▪ High intensity	▪ The intensity	▪ Global minimal path
	▪ Sparseness of the vessel	▪ Distance of the vasculature	
	▪ Smooth in 2D	▪ Curvature in 2D	
	▪ Continuity in 3D	▪ Continuity in 3D	
Zhou et al. [32]	▪ Sparseness of the vessel	▪ Bronchus segmentation	▪ Voronoi division algorithm
	▪ Match with bronchus tree structure	▪ Vessel segmentation	▪ Laplacian filter
	▪ Fissure appearance of line at 2D slice		

### Algorithms based on fissure segmentation

Pu *et al*. have developed an automated fissure detection scheme using a computational geometry approach. The marching cubes algorithm, Laplacian smoothing and extended Gaussian image pyramids are applied to enhance the surface shaped structure within the lung volume [14]. Finally, implicit surface fitting using Radial Basis Functions (RBF) is adopted to segment the lobes [15]. This scheme reduces the dependence on anatomical knowledge and other underlying assumptions, and is less sensitive to noise. As such the integrity of the pulmonary fissure can be assessed [16]. On the other hand, its primary limitation is that some plane-like structure resulting from diseases may be incorrectly considered as the fissure.

Van Rikxoort *et al*. on the other hand have presented a pattern recognition approach, using a supervised fissure enhancement filter [17]. In the training stage, 57 features (40 from several Gaussian filters at different scales, and 12 derived from the Hessian matrix) are calculated for each voxel after which a classifier is trained. Next, a multiphase supervised filtering is executed. This approach gives better fissure detection results than the Hessian matrix filter alone, at the cost of higher computational complexity and does not extend well to pathological cases (e.g. fibrosis). Utilizing the fissure appearance of a curvilinear line, Wei *et al*. have proposed an algorithm including the adaptive fissure sweeping and wavelet transform [18].

An interactive lobe segmentation algorithm has been proposed by Ross *et al*., in which a handful of points are given by the user, and then thin plate splines (TPS) is employed to interpolate a minimally curved fissure surface [19]. This method is later extended to an automatic method using particles, thin plate splines, and maximum a posteriori estimation [20]. Computational complexity however, makes this solution less practical.

Wang *et al*. have introduced a curve growing algorithm modeled by a Bayesian network, which is influenced by the image data and prior shapes of the fissure [21]. They replaced the original image by the ridge map in [22]. However, both the approaches require manual initialization.

### Algorithms based on anatomical knowledge

At the University of Iowa, several lung lobe segmentation algorithms have been proposed. An atlas-driven method is used to find the oblique fissure, in which a fuzzy reasoning system is employed to search the fissure by the combined information from the ridgeness image intensity, smoothness, and the atlas-based search initialization [23]. The algorithm however often yields incorrect results on subjects with unusual anatomy and pathology. Ukil *et al*. used information acquired from airway and vascular tree segmentations to get an approximate region of interest for the fissures, which are further refined by 3D optimal surface detection [24]. For incomplete fissures, incorrect initial lobar segmentation may occur in this algorithm.

In the works performed by Van Rikxoort *et al*., a multi-atlas lobe segmentation algorithm using the lung borders, airways and fissures was proposed to improve the robustness and cope with the incomplete fissures [25-27]. Recently Wei *et al*. developed a new approach with three stages: (a) texture analysis based on a neural network classifier and gray-level run length matrix texture features to localize the fissure region; (b) fissure region analysis by projections resulting; and finally (c) fissure identification by dynamic programming to get the optimal path [28].

Several methods have been introduced by the team from Fraunhofer MEVIS. After combining the 3D Euclidean distance transform image is derived from the blood vessel mask and the gray-level image, a multi-dimensional Interactive Watershed Transform (IWT) is applied to segment the fissures [29]. Four features including the original data from which the blood vessels are removed, the vasculature, the bronchial tree, and the pulmonary fissures enhanced by Hessian matrix based filters are extracted to calculate a cost image, and the watershed transformation is performed to lobar partitioning and classification [30].

By minimizing a 2D energy function on the sagittal slice based on the intensity of the original image, the distance from the vasculature, the curvature in 2D, and the continuity in 3D, Appia *et al*. found the global minimal path in each slice to detect the fissure semi-automatically [31]. After dividing the lung into five sections by a Voronoi division algorithm based on the bronchus and vessel segmentation, the initial fissure region is determined and a Laplacian filter is adopted to extract the final fissure [32].

From the above literature review, it can be seen that the fissure appearances are often used as direct features, but one cannot solely rely on these due to the fissures’ incompleteness. Other anatomical information such as lung structure, vessel and bronchus structures can play auxiliary role. In the present work, we adopt an adaptive fissure scanning method in two sagittal slices to localize the fissure region. Next Uniform Cost Search (UCS) with a cost function based on the Hessian matrix filtered image is employed to finish the fissure extraction at coronal slices. Finally RBF based interpolation is conducted to finalize the lobe segmentation.

## Methods

### Clinical dataset

All CT data sets used in this study are acquired at Shengjing Hospital, China Medical University (Shenyang, Liaoning Province, China) from 2009 to 2014. Data are acquired on a Brilliance 64 CT scanner from Philips Medical Systems (Best, The Netherlands). The transverse images are reconstructed in a 512 × 512 matrix, the in-plane pixel sizes range between 0.6 and 0.8 mm, and the slice thickness is either 0.67 or 1.0 mm. Fourteen subjects (10 normal, 12 male) aged 40–86 years are chosen to evaluate the proposed algorithm. The X-ray tube voltage is set at 120 kV, while the X-ray current ranges 105–378 mA. Reconstruction filters of YB and L are used for 11 and 3 subjects, respectively.

### Overview of the automatic segmentation of the lung lobes

In the proposed approach, there are four stages to achieve the automated segmentation of lung lobe, namely (1) lung segmentation, (2) fissure detection, (3) fissure extension and (4) lobe segmentation. Firstly, the lung segmentation is performed to limit the search space. Secondly the points near the fissure surface are detected by using an improved adaptive fissure scanning method. Next an implicit fissure surface function is obtained using RBF interpolation. Depending on the evaluation of fissure surface functions, lung lobes are ultimately segmented.

### Lung segmentation

**Figure 1:**
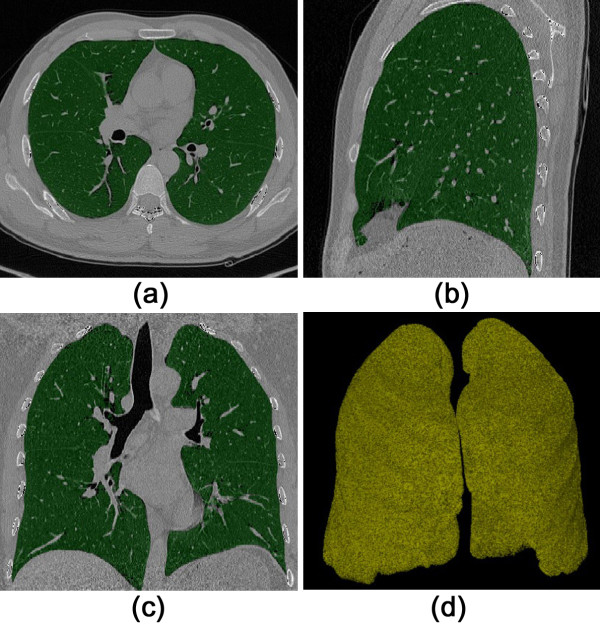
**Lung region segmentation. (a)** transverse slice; **(b)** sagittal slice; **(c)** coronal slice; **(d)** 3D volume rendered result.

A dual-threshold 3D region growing method is adopted to extract the lung regions [33]. The dual thresholds are empirically set to −650 and −930 HU, respectively. Conservative threshold values are chosen to prevent leakage of the segmented space. Next, a closing operation with 7 × 7 kernel is applied on each transverse slice. Finally the tracheal walls and pulmonary vascular structures are discarded from the lung regions by applying a threshold at −300 HU. Representative results are shown in Figure 1.

### Fissure detection

#### Improvement strategy based on lung anatomy

The left lung only has one oblique fissure, while two fissures (horizontal and oblique) can be found in the right lung. The proposed algorithm handles each fissure separately, and the main steps are given as follows.

**Figure 2:**
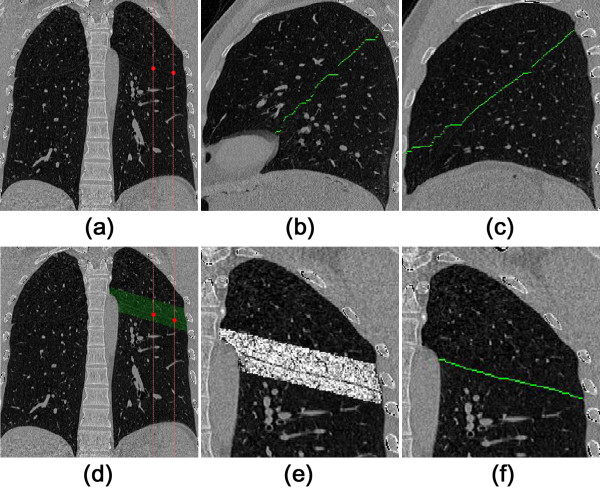
**Improved adaptive fissure scanning procedures. (a)** coronal CT image; **(b)** the first sagittal image slice; **(c)** the second sagittal image slice; **(d)** coronal CT image with *FR* super imposed; **(e)** after Hessian matrix enhancement; **(f)** the final fissure after UCS.

**Step 1** In both lungs two sagittal slices are selected that are sufficiently spaced apart and away from the edge of the lung volume, as shown in (a) of Figure 2. In current algorithm, the two sagittal slices are selected by

(1) $$x_{1}=x_{\text{min}}+0.4\left(x_{\text{max}}-x_{\text{min}}\right)$$

(2) $$x_{2}=x_{\text{min}}+0.6\left(x_{\text{max}}-x_{\text{min}}\right)$$

where *x*_min_ and *x*_max_ are the minimum and maximum *x* coordinate of the segmented lung. The value of 0.4 and 0.6 is set empirically.

**Step 2** The above slides are then processed using a method named Sagittal Adaptive Fissure Scanning (SAFS) as illustrated in next section, to detect the fissure region (*FR*).

**Step 3** Utilizing a line enhancement filter based on the *Hessian Matrix* followed by a *Uniform Cost Search* (UCS), the complete fissure line is extracted from the selected regions as found in Step 2. Results are given in (b) and (c) of Figure 2 as the examples. The line enhancement filter and UCS will be discussed below.

**Step 4** At each coronal slice, there are two marker points generated from the fissure lines as obtained in Step 3. Using these two points, the coronal fissure region is interpolated, as shown in (d) of Figure 2. Similarly, Hessian Matrix enhancement and UCS are employed in this region to get the fissure line in each coronal slice, which are given in (e) and (f) of Figure 2. Hence a set of scattered points is available for further surface extension and interpolation.

#### Sagittal adaptive fissure scanning

**Figure 3:**
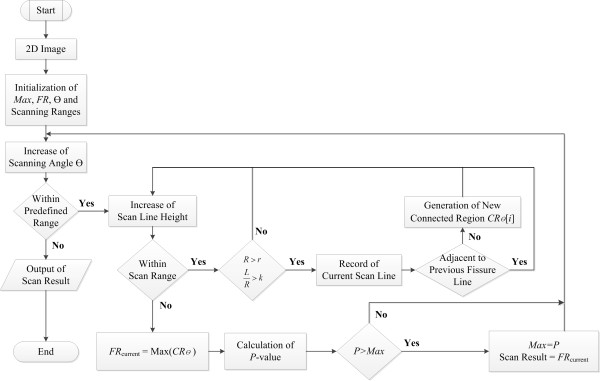
Algorithm flowchart of the sagittal adaptive fissure scanning.

It is well known that the fissure region is devoid of blood vessels and bronchi as it is attached to the boundaries of the two adjacent lobes. Based on this anatomical knowledge, an algorithm is implemented to detect the fissure regions. The method is named the Sagittal Adaptive Fissure Scanning (SAFS), and the flow chart for the algorithm is shown in Figure 3. It an extension of adaptive fissure scanning originating from the reference [18]. The aim of SAFS is to find a region of interest (ROI), i.e., the fissure region, which excludes the blood vessels and bronchi, and thereby models the anatomical assumption about the fissures (no vessels and bronchi in the proximity). The taken approach does not require the full segmentation of the vascular and bronchial trees in a separate step, but implicitly steers clear of these anatomical components.

The algorithm can be divided into three main steps, which are described as follows.

**Figure 4:**
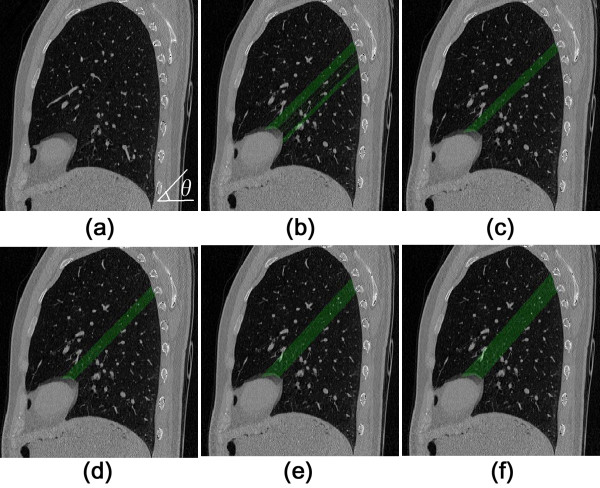
**Sagittal adaptive fissure scanning. (a)** original sagittal CT image; **(b)** multiple connected regions at *θ* = 44.22^o^; **(c)** connected region *CR* with the maximum area at *θ* = 44.22^o^; **(d)***CR* with the maximum area at *θ* = 45.62^o^; **(e)***CR* with the maximum area at *θ* = 46.95^o^; **(f)** The final *FR* with the maximum *P* -value.

**Step 1** Lines are scanned at angles *θ* with respect to the horizontal axis, as can be seen in (a) of Figure 4. The scan line is evaluated to be in the fissure region and is stored only if it meets the requirements of *R* > *r* and *L*/*R* > *k*, where *R* is the length of scan line intersecting with the lung region, and *r* is an empirical value to prevent the selection of lines too short to be part of the fissure, which may occur at the boundary of a lobe. *L* is the supremum of the continuous lengths of the line segments containing pixels with values lower than −970 HU. *k* is another predefined constant denoting the importance of contiguous line segments, and as such modifying the sensitivity of vasculature exclusion. The connected fissure lines form connected regions denoted by *CR*_*θ*_(*i*) for each scan angle *θ*.

**Step 2** One can determine the potential fissure region (*FR*_*θ*_) by finding the largest connected region at each angle *θ,* as shown in (b) and (c) of Figure 4. It means

(3) $$FR_{\theta }=\mathrm{ma}x_{i}\left\{CR_{\theta }\left(i\right)\right\}$$

**Step 3** A score indicating the likelihood of the final *FR* is calculated for each scan angle, and is defined as

(4) $$P=w_{1}N+w_{2}\sigma_{R}+w_{3}\bar{R}$$

where *N*, *σ*_*R*_ and $\bar{R}$ represent the total number of scan lines in a fissure region, the standard deviation of length of scan lines, and the average length of scan lines in a fissure region respectively. In addition, *w*_1_, *w*_2_ and *w*_3_ are the associated weight factors for each parameter. The formulation of *P* is based on the knowledge that the final *FR* should have many scan lines with longer lengths and lower standard deviations of these lengths. Finally the *FR* with the maximum *P*, i.e.,

(5) $$\mathrm{FR}=\arg\;\mathrm{ma}x_{\theta }\left\{P\left(FR_{\theta }\right)\right\}$$

is selected out from the group with different angles *θ*, as shown in (d-f) of Figure 4. The selected regions together are extended such that they form a volume going through both selected slides, in which the fissures can be found.

#### Hessian matrix and uniform cost search

The fissures show up as vague lines in the selected regions from the sagittal images, with intensities only slightly higher than the background of the lung. To increase the probability of success, the lines are enhanced using a Hessian based line enhancement filter developed by Frangi *et al*. [34]. This filter is based on the principle that the eigenvalues of the Hessian matrix denote the curvature of the local image structure. The curvatures on the fissure line are close to zero along the fissure and highly negative perpendicular to the fissure, where the eigenvectors of the Hessian denote the directions of these curvatures.

Using the fissure enhanced image, a cost-function is defined as *C* = *Max*(*E*) − *E*, where *E* is the enhanced image. In this cost function, the fissure is given by the shortest path from one side to the other side, and is found using *Uniform Cost Search* (UCS). UCS is a traditional tree searching algorithm for finding the shortest path between two points in a graph. The image is represented as a graph where all pixels are connected using 8-connectivity, and the cost associated with each edge is given by the value of the destination pixel in the cost function. A point on the inner edge of the candidate region is chosen as a root point, while all points on the edge of the candidate region on the outer side are considered destination nodes. The shortest path is found by continuously expanding those nodes with the lowest cost, keeping track of the direction to go for the lowest cost movement. This process is continued until a destination node is found. After traversing all points on the inner edge, the shortest path between inner and outer edges is obtained finally.

### Fissure interpolation

The proposed algorithm applies Radial Basis Functions (RBF) to do fissure interpolation based on the point set that was found above. Below, the procedure is explained in detail.

#### Scattered point set construction

(a) **Surface points**

After UCS on each of the sagittal slices, a large set of potential fissure points, *PFP*, is generated. It is infeasible to use the whole set for the interpolation of the fissure surface due to its size. From *PFP* a subset *PFP*_*sub*_ is selected in which the points are spaced at least 30 pixels apart in both *x* and *y* directions.

**Figure 5:**
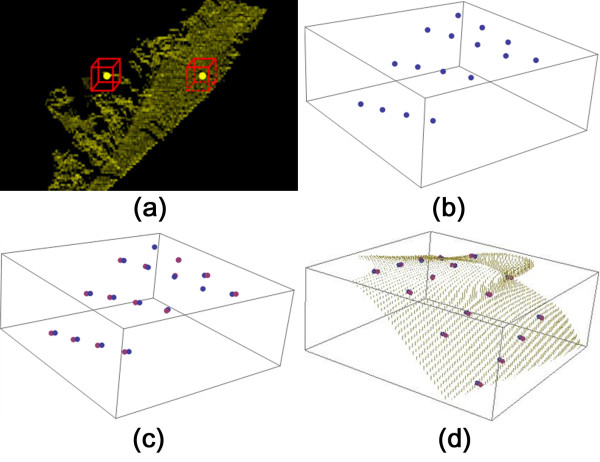
**Illustration of each step in the fissure extension. (a)** surface point verification; **(b)** surface points; **(c)** surface points and off-surface points; **(d)** surface extension.

A cube with size of 11 × 11 × 11 is resampled around each point in *PFP*_*sub*_. If the number of potential fissure points in this cube is larger than 80, the point is stored for further processing. As shown in (a) of Figure 5, the left point is discarded for its cube contains too few scattered points and it may be not reliable. The final *PFP*_*sub*_ is illustrated as in (b) of Figure 5.

(b) **Off-surface point**

For each point in *PFP*_*sub*_, a normal vector can be calculated for the plane spanned by two arbitrary points in the cube and its center point. From the set of all possible normal vectors in this cube, an average normal vector is computed. Finally an off-surface point is determined along the average normal vector at distance *d* = 10 pixels away from the center point. All off-surface points are stored in the set *OSP,* as shown in (c) of Figure 5.

#### Implicit fissure surface function

Implicit fissure surface fitting follows the methods introduced by Pu *et al.*[15]. For completeness, the idea and main steps are briefly presented here. The surface can be estimated using RBF

(6) $$F\left(x\right)=\sum_{i=1}^{n}\lambda_{i}\Phi \left(\left|x-x_{i}\right|\right)+P\left(x\right)$$

where *Φ* is the RBF, *λ*_*i*_ are real number coefficients, *P* is a low degree polynomial, and {x_*i*_} are the scattered points. Here a tri-harmonic spline is selected to be RBF, *Φ* = |x|^3^. *P*(x) is represented in a linear form, *P*(x) = *c*_0_ + *c*_1_*x* + *c*_2_*y* + *c*_3_*z*. For the *PFP*_*sub*_ with *n* points, *F*(x_*i*_) = 0; for the *OSP* with *k* points, *F*(x_*i*_) = *d*.

Hence, Equation (6) can be rewritten as

(7) $$\left[\begin{array}{cccc} \Phi_{11} & \Phi_{12} & \cdots & \Phi_{1m}\\ \Phi_{21} & \Phi_{21} & \cdots & \Phi_{21}\\ \vdots & \vdots & \cdots & \vdots \\ \Phi_{m1} & \Phi_{m2} & \cdots & \Phi_{\mathrm{mm}}\\ 1 & 1 & \cdots & 1\\ x_{1} & x_{1} & \cdots & x_{m}\\ y_{1} & y_{2} & \cdots & y_{m}\\ z_{1} & z_{2} & \cdots & z_{m} \end{array}\;\begin{array}{cccc} 1 & x_{1} & y_{1} & z_{1}\\ 1 & x_{2} & y_{2} & z_{2}\\ \vdots & \vdots & \vdots & \vdots \\ 1 & x_{m} & y_{m} & z_{m}\\ 0 & 0 & 0 & 0\\ 0 & 0 & 0 & 0\\ 0 & 0 & 0 & 0\\ 0 & 0 & 0 & 0 \end{array}\right]\;\left[\begin{array}{c} \begin{array}{c} \begin{array}{c} \lambda_{1}\\ \lambda_{2} \end{array}\\ \begin{array}{c} \vdots \\ \lambda_{m} \end{array} \end{array}\\ \begin{array}{c} \begin{array}{c} c_{0}\\ c_{1} \end{array}\\ \begin{array}{c} c_{2}\\ c_{3} \end{array} \end{array} \end{array}\right]=\left[\begin{array}{c} \begin{array}{c} \begin{array}{c} v_{1}\\ v_{2} \end{array}\\ \begin{array}{c} \vdots \\ v_{m} \end{array} \end{array}\\ \begin{array}{c} \begin{array}{c} 0\\ 0 \end{array}\\ \begin{array}{c} 0\\ 0 \end{array} \end{array} \end{array}\right]$$

where *Φ*_*ij*_ can be determined by

(8) $$\Phi_{\mathrm{ij}}=\Phi \left(x_{i}-x_{j}\right)={\left[{\left(x_{i}-x_{j}\right)}^{2}+{\left(y_{i}-y_{j}\right)}^{2}+{\left(z_{i}-z_{j}\right)}^{2}\right]}^{3/2}$$

with *i*, *j* ∈ [1, *m*],  *m* = *n* + *k*.

Through Doolittle decomposition [35], Equation (7) can be solved, and the parameters *λ* and *c* are obtained. An example of a reconstructed surface is illustrated in (d) of Figure 5.

Finally, three implicit surface functions, *F*_1_(*X*) for the oblique fissure in the left lung, *F*_2_(*X*) for the oblique fissure in the right lung, and *F*_3_(*X*) for the transverse fissure in the right lung are constructed.

### Lung lobe segmentation

After the three implicit fissure surface functions are reconstructed through RBF extension, all points in each of the lungs will be classified using these functions to determine which lobe they belong to. The criteria are shown in Table 2.

**Table 2:** Conditions for determining lobe membership

**Conditions**	**Point location**
*F*_3_(*x*_*i*_) > 0	Right upper lobe
*F*_2_(*x*_*i*_) > 0 ∧ *F*_3_(*x*_*i*_) < 0	Right intermediate lobe
*F*_2_(*x*_*i*_) < 0 ∧ *F*_3_(*x*_*i*_) < 0	Right lower lobe
*F*_1_(*x*_*i*_) > 0	Left lower lobe
*F*_1_(*x*_*i*_) < 0	Left upper lobe

### Accuracy evaluation of lung fissure extraction

To quantitatively evaluate the accuracy of the algorithm, the surface pattern evaluate method, i.e., the shortest Euclidian distance, is adopted. The shortest Euclidian distance from pixel *i* on the manually-defined surface to the surface defined by the algorithm can be computed as

(9) $$d_{i}=\min_{j}\left\{\sqrt{{\left(x_{j}^{A}-x_{i}^{M}\right)}^{2}+{\left(y_{j}^{A}-y_{i}^{M}\right)}^{2}+{\left(z_{j}^{A}-z_{i}^{M}\right)}^{2}}\right\}$$

where $\left(x_{j}^{A},y_{j}^{A},z_{j}^{A}\right)$ and $\left(x_{i}^{M},y_{i}^{M},z_{i}^{M}\right)$ are the coordinates of the voxel on the fissure surfaces obtained by the algorithm and manual tracing, respectively. Correspondingly the mean, standard deviation and the maximum value of *d*_*i*_ can be determined. Moreover, the root mean square (RMS) of *d*_*i*_ is also given as

(10) $$d_{\mathrm{RMS}}=\sqrt{\frac{\sum_{i=1}^{l}d_{i}^{2}}{l}}$$

where *l* is the total number of voxels on the fissure surface by manual tracing.

For each dataset, the fissure contours are drawn by one experienced radiologist slice by slice using the free software of ImageJ. They form the manually-defined surface used as the reference of accuracy evaluation.

## Results

### Fissure detection

**Figure 6:**
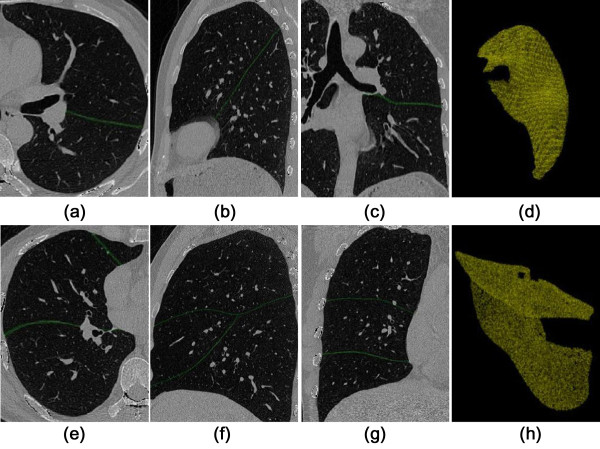
**Fissure detection results. (a)** axial view for left lung. **(b)** sagittal view for left lung; **(c)** coronal view for left lung; **(d)** 3D view for left lung; **(e)** axial view for right lung; **(f)** sagittal view for right lung; **(g)** coronal view for right lung; **(h)** 3D view for right lung.

The proposed algorithm can detect the fissure surfaces successfully for the 14 cases in the dataset. As an example, representative results for one heath subject are presented in Figure 6. For the left lung, the detected fissure curves are accurate in axial, sagittal and coronal views, while the oblique fissure appears continuous and smooth. For the right lung, transverse fissure can be extracted precisely, which traditionally is a difficult task. It can be seen that the transverse and oblique fissures crossover is identified accurately.

**Figure 7:**
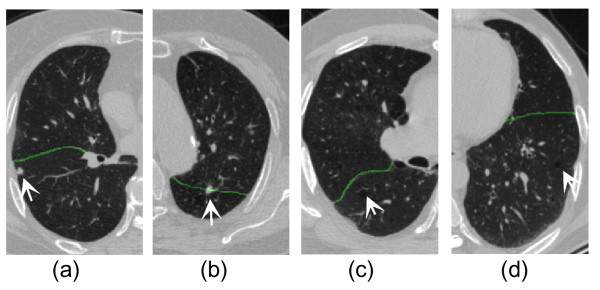
**Fissure detection results for four datasets with lung pathologies (indicated by the arrows). (a)** with a small lung nodule nearby the fissure; **(b)** with a small lung subpleural nodule; **(c)** with centrilobular emphysema with tuberculosis in both apexes; **(d)** with centrilobular emphysema.

Moreover, the current algorithm is used to some cases with lung pathologies. As shown in (a) of Figure 7, the fissure can be acquired for the data with a small lung nodule nearby the fissure. Even with a small lung subpleural nodule illustrated in (b) of Figure 7, the algorithm works well for the RBF interpolation can correct some small errors in fissure detection. Two clinical data sets with mild emphysema are used to assess the proposed method, and the results are given in (c) and (d) of Figure 7. It can be seen the fissures are obtained successfully since the mild emphysema does not change the feature of fissures.

The calculated evaluation measures including the mean, standard deviation, RMS and the maximum value of *d*_*i*_ are given in Table 3. For the right oblique fissure, the mean (±S.D.), RMS, and the maximum averaged over all 14 datasets are 2.05 ± 1.80, 2.46 and 7.34 mm, respectively. The measures are 2.77 ± 2.12, 3.13 and 7.75 mm for the right horizontal fissure, 2.31 ± 1.76, 3.25 and 6.83 mm for the left oblique fissure. For the accuracy of the algorithm, there is no significant difference between the 10 health datasets and 4 pathological.

**Table 3:** The accuracy of fissure detection

**No.**	**Right oblique fissure**			**Right horizontal fissure**			**Left oblique fissure**		
	**Mean ± S.D. (mm)**	**RMS (mm)**	**Max (mm)**	**Mean ± S.D. (mm)**	**RMS (mm)**	**Max (mm)**	**Mean ± S.D. (mm)**	**RMS (mm)**	**Max (mm)**
1	1.53 ± 1.64	2.24	9.83	2.20 ± 1.33	2.57	6.75	2.12 ± 1.09	2.39	4.87
2	1.48 ± 1.27	1.96	4.96	3.19 ± 2.67	4.18	6.23	2.30 ± 2.11	2.65	5.12
3	1.81 ± 1.82	2.57	7.67	2.06 ± 2.83	3.50	11.19	3.16 ± 1.97	3.73	8.83
4	2.30 ± 2.72	2.23	7.36	5.29 ± 3.00	3.95	7.92	1.69 ± 1.17	2.06	4.27
5	2.16 ± 1.57	2.17	6.41	2.92 ± 1.57	3.31	6.72	3.06 ± 3.28	4.48	10.20
6	2.42 ± 1.92	2.61	5.60	1.84 ± 1.66	2.47	6.47	1.63 ± 0.77	1.81	3.28
7	2.28 ± 1.90	2.62	8.40	2.36 ± 0.94	2.00	4.49	2.18 ± 1.71	2.42	6.37
8	2.26 ± 1.63	2.79	7.88	3.25 ± 1.76	2.59	7.59	1.54 ± 1.03	1.85	4.27
9	1.81 ± 1.57	2.40	6.31	2.29 ± 3.27	3.99	13.57	5.68 ± 3.53	6.68	12.44
10	1.85 ± 1.46	2.36	8.56	3.13 ± 2.54	4.03	9.17	1.54 ± 1.87	4.54	5.38
11	2.32 ± 2.25	2.94	8.83	2.35 ± 2.14	3.40	6.85	1.98 ± 1.65	2.34	6.54
12	2.45 ± 1.89	2.95	7.65	2.12 ± 1.29	2.56	6.16	2.14 ± 1.23	3.28	5.49
13	1.92 ± 1.84	2.15	6.35	3.41 ± 2.58	3.19	8.15	1.49 ± 1.52	2.47	8.10
14	2.13 ± 1.67	2.48	6.91	2.38 ± 2.10	2.09	7.31	1.87 ± 1.67	4.86	10.47
Ave.	2.05 ± 1.80	2.46	7.34	2.77 ± 2.12	3.13	7.75	2.31 ± 1.76	3.25	6.83

### Lung lobe segmentation

**Figure 8:**
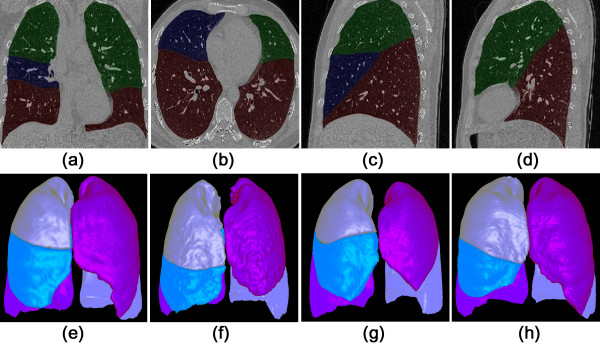
**Results of automatic lung lobes segmentation. (a)** coronal view; **(b)** axial view; **(c)** sagittal view; **(d)** the second sagittal view; **(e-h)** four cases shown in 3D surface rendering.

For each patient, the five lobes are accurately segmented by three implicit fissure surface functions. Figure 8 shows one example in which the lobes are marked with different colors.

The volume of each lobe (cm^3^) and its ratio to total lung volume can be easily calculated according to the segmentation, as summarized in Table 4. This algorithm may be used to do morphometric analysis or functional assessment for the individual lobes [36].

**Table 4:** **Volume of lung lobe (cm**^**3**^**) and the percentage of the volume of each lobe in the whole lung volume (%)**

**No.**	**L-U cm**^**3 **^**(%)**	**L-L cm**^**3 **^**(%)**	**R-U cm**^**3 **^**(%)**	**R- I cm**^**3 **^**(%)**	**R- L cm**^**3 **^**(%)**	**Sum cm**^**3**^
1	1183 (23.6%)	1167 (23.2%)	852 (17.0%)	478 (9.5%)	1339 (26.7%)	5019
2	1597 (21.2%)	1989 (26.4%)	1593 (21.1%)	678 (9.0%)	1680 (22.3%)	7537
3	1027 (24.0%)	857 (20.0%)	914 (21.4%)	399 (9.3%)	1080 (25.3%)	4277
4	1447 (23.4%)	1473 (23.8%)	1233 (19.9%)	492 (8.0%)	1538 (24.9%)	6183
5	1241 (22.5%)	1440 (26.1%)	1050 (19.1%)	488 (8.9%)	1290 (23.4%)	5509
6	1099 (22.1%)	1159 (23.3%)	913 (18.3%)	486 (9.8%)	1318 (26.5%)	4975
7	1292 (22.7%)	1344 (23.6%)	1273 (22.3%)	613 (10.8%)	1174 (20.6%)	5696
8	1599 (23.5%)	1611 (23.7%)	1352 (19.9%)	585 (8.6%)	1647 (24.3%)	6794
9	1284 (24.0%)	1129 (21.1%)	1114 (20.9%)	398 (7.5%)	1414 (26.5%)	5339
10	1021 (25.9%)	691 (17.5%)	858 (21.7%)	390 (9.9%)	988 (25.0%)	3948
11	1412 (25.4%)	1322(23.8%)	1011 (18.2%)	562 (10.1%)	1258 (22.6%)	5565
12	1158 (20.2%)	1402 (24.4%)	965 (16.8%)	489 (8.5%)	1723 (30.0%)	5737
13	1167 (26.5%)	860 (19.5%)	1038 (23.6%)	301 (6.8%)	1040 (23.6%)	4406
14	1311 (28.4%)	966(20.9%)	992 (21.5%)	516 (11.2%)	829 (18.0%)	4614
Ave.	23.8%	22.7%	20.1%	9.1%	24.3%	100%

L-U, left upper lobe; L-L, left lower lobe; R-U, right upper lobe; R-I, right intermediate lobe; R-L, right lower lobe.

After the lobe segmentation, we set the threshold cut-off for emphysema as −950 HU [9], and calculated the percentage of low attenuation area (LAA%) to total lung volume (at −600 HU threshold), i.e., emphysema index, for each lobe in the datasets 13 and 14. For dataset 13, it is 3.14% (the left upper), 0.49% (the left lower), 8.33% (the right upper), 0.66% (the right intermediate) and 0.48% (the right lower). For dataset 14, it is 7.97%, 8.67%, 2.61%, 1.49% and 0.16%, respectively. The potential application is to clinically assess emphysema heterogeneity, find predominantly emphysema lobe and help plan the lung-volume–reduction surgery [11].

### Advantages

One of important advantages of the proposed algorithm is its high speed. Using a desktop PC running Intel Core2 Duo CPU E7500 at 2.0 GHz and 2 GB of memory, to finish the process of lung segmentation, fissure detection, and fissure extension for the dataset with 320 slices of 512 × 512 pixels only takes around 50 seconds. Comparing to the running time of 2.35 minutes [22], 6–8 minutes [23,25], and about 40 minutes [20,32], the current algorithm shows high calculation efficiency. The robustness of the algorithm is preliminarily proved for it works well for the 14 datasets, including two clinical datasets with small nodules and two with mild emphysema.

## Discussions

Adaptive fissure scanning was introduced by Wei *et al.*[18]. In the present study, the idea of adaptive fissure scanning is adopted, but manipulation processes are different. The most important difference in this work is the application of anatomical knowledge to extract the potential fissure regions in lungs. Hence, the scanning only occurs twice to provide the candidate points for the coronal fissure regions determination, which makes the whole algorithm more efficient. At the same time, it also reduces potential fissure incompleteness near the boundaries which often occurs in adaptive fissure scanning.

**Figure 9:**
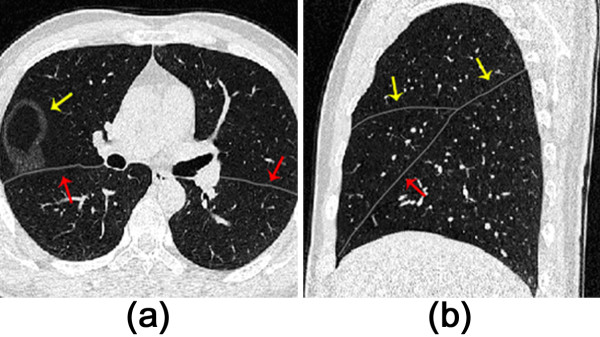
**Determination of the fissure scanning section. (a)** axial view; **(b)** sagittal view.

At the axial sections, the shape of horizontal fissure in the right lung varies significantly, which makes the fissure detection very difficult. For example, the horizontal fissure looks like a circle in (a) of Figure 9. Hence the present algorithm conducts the fissure scanning at the sagittal view. The fissures are more regular than those within the axial sections, as shown in (b) of Figure 9. Takahashi *et al.*[13] consider the assessment of fissure using sagittal images to be more accurate than using axial images. Ukil *et al.*[24] trace the horizontal fissure on the sagittal view for it gives the best contrast. Moreover the work done by Lassen *et al*. also shows that the best segmentation can be achieved in sagittal orientation [30].

Because fissures are often incomplete and barely visible in CT images, fissure interpolation is required. Surface fitting based on RBFs using tri-harmonic splines, is used here. One of the advantages of RBF surface interpolation is its high speed, taking less than a second in the present implementation. The quality of the lobe segmentation is strongly influenced by the number of the input points obtained from fissure detection.

For accuracy evaluation of fissure detection algorithm, the measures based on the shortest Euclidian distance have been adopted by most references [14,17-25,31,32,36]. The mean (±S.D.), RMS, and the maximum are 2.77 ± 2.12, 3.13 and 7.75 mm for the right horizontal fissure in present study. Given different datasets, current accuracy reaches the same level as the state-of-art works. For example, RMS (±S.D.) is 2.04 ± 3.88 [14], 2.23 ± 2.52 [20], 1.96 ± 0.71 [23] and 2.5 mm [31].

Current algorithm shows good robustness for the 14 datasets, including two clinical datasets with small nodules and two with mild emphysema. Hence, the potential applications of the proposed algorithm might include but not limited to the morphometric analysis for the lung lobe volume, functional assessment of the individual lobe [36], locating the lung nodules into the lobe [25] embedded into computed aided detection system of lung cancer, and quantitative assessment of emphysema index for each lobe [9,11], etc.

Our study has several limitations. Due to the dependence on the anatomical knowledge, the present algorithm works well for the regular major fissures, but is not well suited for the accessory fissures. At the same time it also does not deal well with the irregular fissure morphologies, especially circular fissure. Furthermore, the accuracy of fissure detection is rather low for the regions near the lobe edges for two reasons. Firstly, straight lines are used to approximately determine the fissure region, which results in missing parts of the fissure lines. Secondly, some unreliable scattered points may lead to a wrong implicit surface function.

Only 14 datasets are tested for our methods. Two datasets with small nodules and two with mild emphysema are too few to prove the robustness fully. The current method cannot cope with the lung with heavy diseases, such as the advanced tumor and interstitial lung disease, for the fissures appear very irregular. The interactive operations must be followed.

As far as the authors know, there is no open-source code for the fissure detection and lobe segmentation. Due to time limitations we were unable to implement the other methods, but instead have chosen to compare the performance of our method to previously proposed methods in literature [14,17-25,31,32,36]. In general, more validation and comparison will be conducted in the future working.

Though the adaptive strategy is employed, a few parameters have to be determined empirically, and might be not suitable for other datasets. For example, two sagittal fissures are determined by Equations (1) and (2), which is essential to the success of the present algorithm. Any incorrect detection will cause the failure of the lung lobe segmentation. In SAFS, *r* is set as 30–80, the optimal value is 50. *k* can range from 0.4 to 0.7, the optimal value is 0.6, which works well for all 14 subjects in the present study. SAFS is the vital step in the algorithm, and to verify its results before continuing other parts is recommended. Fortunately, it is easy to judge for only two sagittal slices are involved. For radial basis interpolation, the point space is 30 voxels, and the threshold of 80 potential fissure points within a 11 × 11 × 11 cube is adopted to ensure the resampled point is reliable. The distance between the center point and the off-surface point *d* is set to be 10. Because of the interpolation, these parameters are not very sensitive to the accuracy of algorithm.

The simplified lung segmentation in current study may not work for the other data with severe lesions. Considering the main goal of this paper is to detect the fissure and extract each lobe, we did not implement more sophisticated methods, but more advanced methods [37,38] can be used to replace the existing method.

## Conclusions

An automatic fissure detection and lobe segmentation algorithm is developed and evaluated on fourteen CT scans. It is found that sagittal adaptive fissure scanning can localize the potential fissure regions quickly, using knowledge on the density of blood vessels and bronchi. Once the potential region is enhanced by a Hessian based line enhancement filter, Uniform Cost Search (UCS) can extract the fissures successfully in 2D. Furthermore, surface fitting based on RBFs is able to obtain three implicit surface functions for each dataset. The current algorithm shows good accuracy, speed and robustness through evaluation by 14 datasets including two with small lung nodules and two with mild emphysema. For example, averaged over all datasets, the mean (±S.D.), RMS, and the maximum of the shortest Euclidian distance are 2.05 ± 1.80, 2.46 and 7.34 mm for the right oblique fissure. Lung anatomical knowledge is applied to localize the potential fissure regions, which makes the algorithm fast. The complete segmentation procedure takes less than one minute on a modest desktop PC. The algorithm is robust, enables to deal with the 14 experimental datasets successfully, and may help locate the lesions into each lobe and analyze them regionally.

## Abbreviations

CT: Computed tomography; COPD: Chronic obstructive pulmonary disease; EV: Emphysema volume; EI: Emphysema index; RBF: Radial basis functions; TPS: Thin plate splines; IWT: Interactive watershed transform; UCS: Uniform cost search; SAFS: Sagittal adaptive fissure scanning; RMS: root mean square.

## Competing interests

The authors declare that they have no competing interests.

## Authors’ contributions

SQ: proposed the idea, performed experiments, analyzed the data, made discussions and composed the manuscript together with HJWT, MX. YY: provided CT images, segmented the lobes manually, and made the discussions. YK: directed the experiments and made discussions. All authors read and approved the final manuscript.

## Authors’ information

Shouliang Qi and Han JW van Triest share first authorship.
